# Lectures on Diseases of the Skin

**Published:** 1855-05

**Authors:** Austin Flint

**Affiliations:** Professor of the Theory and Practice of Medicine in the University of Louisville, Ky.


### MEDICINE AND SURGERY.

**May, 1855.** **NEW SERIES.** **Vol. III.—No. 5.**

### Art. I.—LECTURES ON DISEASES OF THE SKIN.

BY AUSTIN FLINT, M. D., PROFESSOR OF THE THEORY AND PRACTICE OF MEDICINE IN THE UNIVERSITY OF LOUISVILLE, KY.

### LECTURE I.

*Gentlemen*:—

Before directing our attention, in this lecture, to some considerations preliminary to treating of the diseases affecting the skin, we will glance, for a moment, at its anatomical composition and functions. Commencing an analysis from the exterior, we find, first, a thin, insensible, horny layer called the epidermis, cuticle or scarf skin. This has been heretofore considered to be an inorganic production—a mere concretion of plastic exudation from the parts beneath. Microscopical researches show it to be resolvable into nucleated cells, and fully entitled to be regarded as an organized tissue. Apparently underlying the cuticle is a substance called the rete-mucosum, the seat of color, and the diversities of complexion. This is sometimes described as a portion of the cuticle or epidermis, and sometimes as a distinct layer of the skin. Microscopically studied, it is found to consist of pigmentary granules contained within the cells of the epidermis, being black in the negro, of different shades of color in the different races, and presenting varying tints in different individuals. It is, in reality, an element of the epidermis.

Lying beneath the cuticle is the dermis. This is divisible into two strata, viz: the papillary, and the corion. The former is of less consistence than the latter, and is arranged in the form of minute papillæ, in which is supposed to reside, *par excellence*, the sense of touch, or feeling. The latter is firmer, areolar in its texture, containing white inelastic, commingled with yellow elastic fibres, the latter, under the influence of cold, and sometimes mental emotions, undergoing sensible contractions, giving rise to the appearance vulgarly known as goose-skin.

In the areolæ of the corion are found cryptal, cœcal, or minute blind cavities in which an unctuous matter is secreted. These are the sebaceous glands or follicles. Beneath the integument, in the subdermoid tissue, are found small convoluted tubuli, or congeries of globular sacs which furnish the special secretion constituting the insensible and sensible perspiration. These are the sudoriparous glands. The tubuli unite to form ducts which, pursuing a serpentine course, pass through the dermis, and terminate by open pores upon the surface of the epidermis. The perspiratory tubuli and glands, although separately considered quite insignificant in size, constitute in their aggregate over the entire surface of the body an immense secreting surface. It is estimated by Mr. WILSON that, thrown into a single canal, the united tubuli would form a duct forty-eight thousand six hundred yards in length, or nearly twenty-eight miles! The average daily loss of matter by means of the secretory function of this glandular element of the cutaneous surface, is estimated to be about two pounds and a half. A large proportion of the matter eliminated is water. Carbonic acid is also thrown off—and a certain amount of nitrogen. The proportion of solids to the whole amount of perspiration is small, not exceeding a few scruples in the twenty-four hours. Still, as a depurative organ, the skin possesses great importance in the animal economy.

The skin is richly supplied with blood-vessels, absorbents and nerves. It therefore has a high degree of vital activity, and is the most sensitive of the compound structures.

The hair and nails are called appendages of the skin. Their composition is similar to that of the epidermis, and they may be considered, in fact, component parts of that portion of the cutaneous system.

To describe minutely the anatomical structure, mode of development and functions of the different cutaneous elements which have just been mentioned, does not, of course, fall within my province. These different elements, however, are, to some extent, individually the seat of disease, and they contribute, separately, to furnish distinctive characters pertaining to different cutaneous affections; hence, we shall have occasion to refer to them in treating of this department of special pathology.

---

When we consider in connection with the complexity of the anatomical composition of the skin, the extent of its expanded surface, its exposure to the direct action of external morbid influences, its high grade of vital activity, its great nervous susceptibility and its important function as an excretory organ, we should naturally infer that it must be liable to various forms of morbid action. Such is the fact. The proportion of cutaneous affections among the variety of diseases which come under the observation of the practitioner of medicine is considerable, and they are presented under a great diversity of aspects. It would also be a natural inference from these circumstances that this class of diseases would occupy not a small share of the attention of medical practitioners, and fill a commensurate space in medical literature. When it is farther considered that these affections, being situated externally, are open to direct examination and study by means of the senses, in this respect offering great advantages over those which, concealed from inspection during life, can only be investigated by means of their signs and symptoms, it might reasonably be expected that our scientific and practical knowledge of them would be more complete, and that it would be more generally diffused among the profession than in the instance of almost any other class in the nosology. The state of the case, however, is the very reverse of this. There are few diseases in the whole nosological catalogue of which science possesses so little satisfactory information, and which are so generally neglected by practitioners, as those affecting the skin. There is scarcely any other class of which so little has been written. The whole bibliography of skin diseases will not embrace many treatises. Systematic writers on practical medicine are accustomed to pass them over with very cursory notice. Formal instruction relating to them rarely enters into the curriculum of instruction in our medical schools; and the practitioner seldom feels mortification in betraying utter ignorance of what has been accomplished in this department of practical medicine. I believe that I do not do injustice to the medical profession when I say that the majority of its members pay little, or no attention to the differential diagnosis of cutaneous diseases. Therapeutical measures are therefore indiscriminately employed, without reference to distinctions, which, in this point of view, are of practical importance. Hence it is that so few have contributed to the general stock of knowledge; and hence, also, is it, in a measure, that the skin affections hold a preëminent station among the *opprobria* of medicine. Of all diseases these are perhaps most apt to persist in spite of remedies, and they are therefore a profitable field of revenue for the charlatan, and the vender of secret nostrums. The inquiry will not fail to arise in your minds, whence this strange neglect of a class of affections which all must admit are by no means least in importance? We shall be better prepared to meet this question hereafter, but I will say here that although it arises, in part, from difficulties inherent in the study, it is attributable, in no small measure, to the fact that the study has been rendered gratuitously complicated. I will enlarge upon this point somewhat presently, and in this connection will therefore only observe, that if the diseases of this class were to be presented with that simplicity which is not only admissible, but fully compatible with the attainment of all important scientific and practical ends, there would be no excuse for the prevalent neglect of which I have spoken, but, on the other hand, this department of special pathology would present to the student and practitioner peculiar attractions.

The great variety in the external characters and other circumstances pertaining to cutaneous diseases, is calculated to give rise to an impression that they are too numerous, diversified and eccentric for either scientific analysis, or practical discrimination. But without some classification based upon resemblances reliable, and recognisable, it is evident their investigation must be exceedingly imperfect and unsatisfactory. The study of this class of diseases may be said to have first become truly a department of medical science when its subjects were first generalized and distributed into a systematic arrangement. This was the origin of dermatology, as the science treating of cutaneous diseases is sometimes styled. The first successful architect of this science was PLENCA, or PLENCK, a Hungarian physician. To him is due the credit of first forming a classification founded on elementary characters appertaining to the various forms of cutaneous affections. Prior to his time they had been distributed by some writers according to the portion of the body affected—the head, trunk and extremities; and by others they were arranged according to their supposed causes, which were too little known to serve as a permanent basis for that purpose. PLENCK published his classification in 1776 (a date which should be easily remembered by the American medical student). In 1798, WILLAN, an English writer, published a treatise in which he adopted PLENCK's classification, with some important modifications. Since the time of the latter publication, the classification has been known as the *Willanean arrangement*, and has been generally accepted, with additional improvement, by the profession in all countries. The classification of PLENCK and WILLAN is founded on resemblances in external characters determinable in the early stages of cutaneous diseases. Notwithstanding these diseases after some continuance present widely different appearances, it was ascertained that in their primitive, elementary forms they are resolvable into a few groups. PLENCK established fourteen classes, or orders. WILLAN reduced this number to eight, which are still recognised by most writers, although it is probable a still greater reduction is admissible. Attempts have been made to substitute other methods of arrangement for the Willanean. The distinguished French dermatologist, ALIBERT, and one of his successors at the St. Louis hospital, CAZENAVE, have made such attempts. But perhaps the ablest effort of this kind is that of an English author, Mr. ERASMUS WILSON. Mr. WILSON endeavors to institute divisions after distinctions founded on the particular anatomical elements of the skin affected, and on supposed intrinsic differences in the nature, as well as seat of the morbid action. Were this plan practicable it would undoubtedly be the most scientific and satisfactory. But we are not, with our present knowledge, prepared to decide on such distinctions; and there is great risk of substituting speculations for facts in distributing the different affections after this plan. We could not discuss the merits of the *Wilsonian* method, as contrasted with the *Willanean*, till the latter has been considered more fully; but it will hardly be advisable to engage in such a discussion. The system of WILLAN appears to my mind far more simple, and in every respect preferable to that of WILSON, or any other so called natural arrangement. It is depreciatingly termed an artificial system, and so to some extent it undoubtedly is. But it is based on positive anatomico-pathological distinctions, which are visible, generally ascertained without great difficulty, involving no hypothetical assumptions, and, practically, it is convenient and trustworthy. The fundamental principles on which it rests possess so many advantages that I do not believe it will very soon, if ever, be supplanted, although, as already remarked, it admits of greater simplification and improvement than it has yet received.

---

The *eight* groups, or orders of the Willanean system are as follows:

*First group or order. Exanthematæ. Rashes.* Most of the affections embraced in this class we have considered under the head of febrile diseases. They constitute the eruptive fevers. They are characterized, each by a peculiar eruption, and their diagnostic criteria consist, in a great measure, of the characters pertaining to this eruption; but the cutaneous affection is confessedly secondary, presenting the external manifestations only of a general disease, viz: *Fever.* We have every reason to believe, it is true, that other groups of skin diseases are equally related to some internal derangement, but the connection has not been so positively established, and too little is known respecting the primary disorder in other classes, to admit of their entering into a system of classification. Treating of the exanthemata in another part of my didactic course, viz: in connection with fevers, I shall not take them up under the head of cutaneous diseases.

*Second group or order. Vesiculæ. Vesicles.* The characteristics of this class are the presence of small elevations of the cuticle, containing a fluid, at first transparent, resembling serum or water. In most instances, after a time, the contained fluid becomes opaque, milky, puruloid or distinctly purulent. The liquid may be resorbed, or discharged by rupture of the vesicles, forming crusts, and sometimes scabs, more or less intermingled with scurf or epidermic scales. Vesicles are always minute in size, each holding a small quantity of serum or lymph.

*Third group or order. Bullæ. Blebs.* This class consists of tumors caused by the elevation of the cuticle from an effusion of serum or sero-purulent fluid, between the epidermis and corion. Their size varies from that of a pea, to an egg. The type of this group is a blister. They resemble, in every particular, excepting size, vesicles; and the two might appropriately enough be embraced in the same order, as has been done by some late writers, (NELLIGAN, CAZENAVE, and BENNETT). Practically, however, this does not simplify the subject, since it is as easy, if not more so, to recollect the bullæ as a distinct order, than as different genera of another group.

*Fourth group or order. Pustulæ. Pustules.* These terms signify pimples containing pus. An elevation of the cuticle containing, *from its development*, a purulent fluid, comes in this class. Pustules rarely end by resorption, but the pus escapes and concretes, forming scabs. Vesicles frequently, during their progress contain purulent fluid, but it is preceded by serum or lymph. Pustules, from the earliest period that the nature of the fluid can be ascertained, are found to contain pus; and herein lies the essential point of distinction.

*Fifth group or order. Papulæ. Pimples.* Pimples are small solid elevations containing no fluid. By their solidity and want of fluid contents, they are sufficiently distinguished from vesicles and pustules, with which alone they can be confounded.

*Sixth group or order. Squamæ. Scales.* The distinguishing characters of this class are thickened, laminated, epidermic scales, which are exfoliated, and successively renewed in greater or less abundance. These can only be confounded with the crusts formed by the desiccation of the effused serum or lymph of ruptured vesicles; but the latter are preceded by a liquid exudation, while the former are, from the first, dry scales, produced by morbid action of the epidermic cells.

*Seventh group or order. Maculæ. Spots.* This is a class not having much practical importance, embracing affections some of which can hardly be considered forms of disease, and in all cases easily distinguished from the diseases belonging to the other classes. They are seated in the rete mucosum, or are occasioned by effusion of blood in minute spots.

*Eighth group or order. Tuberculæ. Tubercles.* Tubercles, to quote the definition of RAYER, are "small, solid, circumscribed, indurated and enduring tumors, which, after continuing some months, often for several years, almost uniformly end by becoming changed in their nature and falling into a state of ulceration." From this brief description it is plain they need seldom, if ever, be confounded with the diseases belonging to other classes. In general, they are of infrequent occurrence save in tropical climates. The forms occasionally presented with us are treated of in the department of surgery under the head of tumors. For this reason I shall not take up the affections falling under this class.

From the foregoing eight groups or orders, eliminating two, viz: Exanthematæ and tuberculæ, there will remain six to be considered respectively, viz: Vesiculæ, Bullæ, Pustulæ, Papulæ, Squamæ and Maculæ. I shall take them up successively in the order just enumerated.

From the brief notice of the distinctive features of each of the several classes just enumerated, you will, I think, perceive that, so far, the arrangement is sufficiently simple, and that to determine in practice to which of the classes individual cases belong cannot generally be a very difficult undertaking. I shall present the characteristic circumstances appertaining to each class at greater length as I take up the respective groups in succession. The object in already referring to the more prominent of the distinguishing features is, that you may form an idea of the different classes collectively, before considering each class separately.

These several groups or orders are subdivided into genera and species. Here the classifications of WILLAN, and of all writers who have cultivated dermatology as a speciality, present a complexity which gives to the study a formidable and forbidding aspect. It is owing to this, in a great measure, that the subject is so generally neglected. It is clear to my mind that there is an over refinement in the minute subdivisions, and the technical distinctions of WILLAN, and most other dermatologists; and inasmuch as upon this point it hinges, whether the medical student will decide to make himself master of the subject, or turn away from it as an impracticable branch of medical education, I may be excused for dwelling a little upon it. While I do not doubt that the study of diseases of the skin, like other medical specialties, affords ample range for exclusive industry, and admitting that to prosecute this department of practical medicine in all its minutiæ, ordinary facilities of observation are inadequate, I wish nevertheless to assume the position that, with such a proportionate degree of attention as will not necessarily compromise due proficiency in other branches of equal claims, and with the opportunities accessible in all large towns, a very satisfactory degree of acquirement, and of practical skill in diagnosis are attainable. When the student or practitioner opens a voluminous treatise, like that of RAYER, for example, devoted entirely to skin diseases, he is apt to be repelled by the apparent magnitude of the subject. When he considers, also, that exemplifications of the various forms of disease in prevate practice, or general hospitals, are presented only occasionally, and can only be studied with intervals of more or less duration between their occurrence, he is apt to become discouraged, and imagine that it is in vain to make any effort for personal qualification in this department of medical art, without the advantages of hospitals or dispensaries instituted with especial reference to cutaneous diseases. The very great facilities afforded by such institutions (which exist only in some of the larger metropolitan towns, like Paris) are sufficiently obvious. Very much time and labor are saved by having at the same moment before us illustrations of every variety of the subject which a scientific pursuit embraces. But for many, if not most persons these advantages are not available. The question, then, is, not whether they would be desirable, but whether they are absolutely essential. Shall the subject be neglected because these valuable opportunities are not to be obtained; or may they be dispensed with, and success secured by improving the comparatively few facilities which are available? This is the true question. And my answer is, that with means accessible to all, and with such an amount of exertion as may be bestowed without neglect of other matters, one may qualify himself to understand and treat with satisfaction this division of diseases. It may be said of this subject, as of another branch of practical medicine, I refer to the physical exploration of the chest, that the want of a proper appreciation of the fewness and simplicity of the rules which are really of much practical consequence, constitutes the greatest obstacle to entering on the pursuit. The grand division of skin diseases into groups, or orders, as has been seen, is sufficiently intelligible. A very little study and observation are adequate to the mastery of the subject to that extent. The subdivisions into genera and species are, for ordinary practical purposes, needlessly numerous, especially the multiplication of species. They are refinements which are very well in a purely scientific point of view, but it is not necessary that the student or practitioner shall retain them all in his memory, for the usual duties of medical practice. Let him be prepared to refer different affections to their appropriate orders; let him be conversant with the more prominent generic distinctions, and let him have a general knowledge of those specific differences which are most liable to occur, and he is abundantly qualified to recognise and treat individual cases with a satisfactory degree of success.

The nomenclature of Willan's system is a great evil. The names are pedantic, often barbarous, devoid both of euphony, and of that significance which assists the memory by striking associations. The system admits of great improvement in this respect, but the terms have become so interwoven in medical literature, that it would not be easy to introduce others more simple and appropriate. A great number of them, however, may be practically disregarded without any scruples. I shall have occasion to refer to these views, and to adduce illustrations of their verity, in treating of the particular diseases of this class. I shall be well contented with the fruits of the few discourses which I purpose to devote to the subject, if they simply tend to convince you that there is no validity in the reasons usually assigned for neglecting cutaneons diseases.

---

Before proceeding to take up the individual affections embraced in the several classes, it may be useful to present a few general considerations applicable to the *pathology*, *management* and

*diagnosis* of diseases of the skin. I have already alluded to the fact that, in most of these diseases, (as well, indeed, as in a large proportion of affections of other structures), the true primary seat is not in the part affected—the skin—but the local morbid actions take place in consequence of some prior, internal or general disorder. To quote the expressive language of RAYER, the *roots* of cutaneous diseases generally lie within the system. They belong, in other words, among what it has been customary to call constitutional affections. This term *constitutional* it will be often convenient to employ. I will therefore briefly state what it is intended to denote. Not a few local affections, as they are classified and named in the nosology, in reality depend on morbid conditions other than those which can be seen or studied. They proceed from some general disorder within the economy, which science, as yet has not succeeded, to much extent, in explaining, or even in describing. In many, if not most cases, the fluids are probably at fault. Sometimes we know this to be the fact; but whether the humoral perversions are primary or secondary, is not, in all instances, an easy matter to determine. In our ignorance of the true character and seat of these general disorders, we must content ourselves, for the present, with merely designating them, collectively, by some name which will enable us to refer to the fact of their existence, as occasion may require. *Constitutional* is the title frequently employed for that purpose. We mean then, by a constitutional disease one that involves some inappreciable disturbance in the economy, occurring anterior to the local affection which presents itself to our notice—the nature of the former being frequently either obscure, or utterly unknown. Affections of the skin are, for the most part, constitutional in this sense of the term; and this is a very important fact to be considered, albeit the term conveys so little real information concerning their essential pathological character. In our therapeutical management, and in our efforts to obviate their causes (the latter often being essential to successful treatment), if we do not take into proper consideration their dependence on conditions of the system at large, we shall rarely meet with much success. The larger number of the most effective remedies in these diseases, are those which have some general influence within the economy, or, in other words, remedies which are constitutional in their *modus operandi*. The following points, worthy of consideration in themselves, go to establish the view just presented:

1st. In the great majority of instances cutaneous affections are not referrible to direct, local external causes. This may be said to be true, as a general remark, of these diseases as a class. A few are apparently occasioned by the application of irritating substances, as when, for example, eczema attacks the hands of grocers or bakers. But it is by no means certain but that in instances like these the disease involves a constitutional predisposition, and the locality of the affection alone is determined by the external irritants.

2nd. There are various forms of cutaneous disease, and each form has its own peculiar characters. This in some measure is due to the different anatomical elements entering into the structure of the skin being affected repeatedly; but that ulterior causes are involved, is shown by the fact, that, to some extent, we can trace connections between special forms of disease, and certain conditions of the economy attributable to known causes. Thus a species of pustule called *acne rosacea* occurs usually in persons addicted to the use of spirits; certain forms of vesicular and pustular eruptions affect the face and head of children who are ill-fed and poorly cared for, and also during weaning and dentition.

Were we able to ascertain with precision in particular cases what the general perversions are, of which these cutaneous eruptions are the local expressions, we should understand why it is, that, under different circumstances, different forms of disease are manifested. But, with our present knowledge, we are here almost entirely in the dark. We can study effects, and sometimes trace to remote causes, but the intermediate links of the chain by which the causes and effects are joined, remain to be disclosed by future researches.

3rd. Diseases of the skin are apt to be obstinately persistent. They tend to protractedness in a large proportion of instances, showing that there exist internal, determining causes maintaining their continuance. The latter inference is shown to be rational by contrasting, as regards duration, spontaneous eruptions, as they are called by way of distinction, with those produced artificially by some local irritant like croton oil, or the tartar emetic ointment. The latter usually speedily get well, after the operation of the local cause ceases. That the former do not, is doubtless attributable to the persistence of their internal or constitutional causes.

4th. Eruptive fevers are confessedly constitutional affections. Analogy leads to the conclusion that other eruptions are equally so, on the logical principle that resemblance in effects is presumptive evidence of a similar relation in causation.

5th. It has been already remarked that skin diseases are most frequently cured by remedies which exert some general influence on the economy—an influence called eutrophic, or alterative.

6th. Internal disorders are frequently relieved by the occurrence of eruptions; and, on the other hand, their repercussion either spontaneously, or by means of external medicaments, sometimes occasions internal disorders; showing that a morbific agent, or, at least, a morbific principle has undergone a change in location, or, as it is technically called, a *metastasis.*

7th. The appearance of cutaneous eruptions is frequently preceded by appreciable constitutional symptoms such as chills, febrile movement, disturbance of the digestive system, &c., which disappear, in some instances, so soon as the local affection becomes developed.

8th. Eruptions are produced by eating particular kinds of food, such as shell fish, and by some internal remedies as balsam copaibæ, iodide of potassum. In either case the agency in producing the local affection must be exerted by means of morbid conditions induced within the system.

9th. We have an instance in which various diseases of the skin are known to result from the introduction into the system of a special poison, viz: the syphilitic virus. Several specific forms of eruption proceed from this source, constituting a group which is sometimes distinguished as the syphilides. Syphilitic eruptions pertain to the subject of syphilis, and will therefore come within the province of my distinguished friend and colleague, the professor of surgery.

The foregoing considerations are adduced as establishing conclusively (in the absence of direct demonstrative evidence) the constitutional origin of cutaneous affections in general. I have taken pains to present this summary of the more important of the facts from which this conclusion is logically drawn, because the doctrine is not alone interesting in a pathological point of view, but it is of great practical consequence, and is too often overlooked by practitioners in treating cutaneous diseases. It were much to be desired that science possessed more knowledge than is yet acquired of the essential pathological nature of this class of affections. For the most part, scientific researches have been hitherto limited, of necessity, to the description of their external characters. Dermatology is little more, as yet, than a branch of natural history. Specimens have been analyzed, compared and classified, and these are no mean results. It remains to ascertain upon what different internal conditions they depend. It may be long ere much useful information in this direction will be obtained, yet there is reason to hope they will ultimately be elucidated. I cannot but indulge the expectation that the appliction of analytical chemistry to the study of the fluids in health and disease, will, by and by, shed light on this interesting and important field of inquiry. Many considerations go to sustain the hypothesis that the proximate causes of most of the cutaneous diseases are humoral. It is not worth our while in this connection to discuss the merits of this hypothesis; since we should not be led by such a discussion, to take a decided position with respect to it. The prudent course is to preserve the mind sufficiently unbiased to be ready to receive facts and deductions as they are developed in the progress of science, and subject them to an unprejudiced examination.

The therapeutical course in general to be pursued in treating diseases of the skin, must be considerably influenced by the pathological views which have just been expressed. We are always to look to the constitution, obviating remote causes which affect it unfavorably, reforming habits, and improving its condition according to the indications which may be presented. We cannot always apply rational measures to the treatment of diseases of the skin, because we do not know the actual nature of the interior morbid perversions from which they spring. We can only follow what imperfect light we have. As regards the remedies to be employed, it follows from the views which are here presented, and it will be seen hereafter to be the fact, that generally their supposed efficacy has been established empirically, that is to say, based on experience exclusively, not on any rational explanation which we are able to offer of the immediate effects which they produce.

